# Expanding the Phenotype of B3GALNT2-Related Disorders

**DOI:** 10.3390/genes13040694

**Published:** 2022-04-14

**Authors:** Erika D’haenens, Sarah Vergult, Björn Menten, Annelies Dheedene, R. Frank Kooy, Bert Callewaert

**Affiliations:** 1 Center for Medical Genetics, Ghent University Hospital, 9000 Ghent, Belgium; erika.dhaenens@ugent.be (E.D.); sarah.vergult@ugent.be (S.V.); bjorn.menten@ugent.be (B.M.); annelies.dheedene@ugent.be (A.D.); 2 Center for Medical Genetics, Antwerp University Hospital, 2650 Edegem, Belgium; frank.kooy@uantwerpen.be

**Keywords:** *B3GLANT2*, dystroglycanopathies, congenital muscular dystrophy, muscle–eye–brain disease, Walker–Warburg syndrome, Fukuyama congenital muscular dystrophy, uniparental disomy, whole-exome sequencing

## Abstract

Dystroglycanopathies are a group of congenital muscular dystrophies (CMDs) that include a broad phenotypic spectrum ranging from late-onset limb-girdle muscular dystrophy to severe muscle–eye–brain disease, Walker–Warburg syndrome, and Fukuyama congenital muscular dystrophy. In addition to clinical heterogeneity, CMDs are characterized by genetic heterogeneity. To date, 18 genes have been associated with CMDs. One of them is *B3GALNT2*, which encodes the β-1,3-N-acetylgalactosaminyltransferase 2 that glycosylates α-dystroglycan. In this study, using exome sequencing, we identify a homozygous frameshift variant in *B3GALNT2* due to a mixed uniparental disomy of chromosome 1 in a 7-year-old girl with global developmental delay, severely delayed active language development, and autism spectrum disorder but without any symptoms of muscular dystrophy. In addition to this case, we also provide an overview of all previously reported cases, further expanding the phenotypic spectrum.

## 1. Introduction

The dystrophin glycoprotein complex (DGC) is a large complex of glycoproteins anchoring the cytoskeleton to the extracellular matrix, and it plays a major role in the development and function of muscle, nerves, the heart, the eyes, and the brain [1,2]. α- and β-dystroglycans are components of the DGC and are encoded by the dystroglycan gene (DAG1, OMIM *128239). The encoded precursor protein is posttranslationally cleaved into α- and β-dystroglycans (α-DG and β-DG). α-DG is an extracellular glycoprotein that binds to extracellular matrix proteins, such as laminin, perlecan, biglycan, and neurexin, and it binds to transmembranal β-DG. β-DG, however, binds the intracellular dystrophin that interacts with the cytoskeleton. α-DG and β-DG undergo differential glycosylation in muscle versus non-muscle tissue. The correct O-glycosylation of α-DG is essential for its function as an extracellular matrix receptor [3,4].

**Figure 1:**
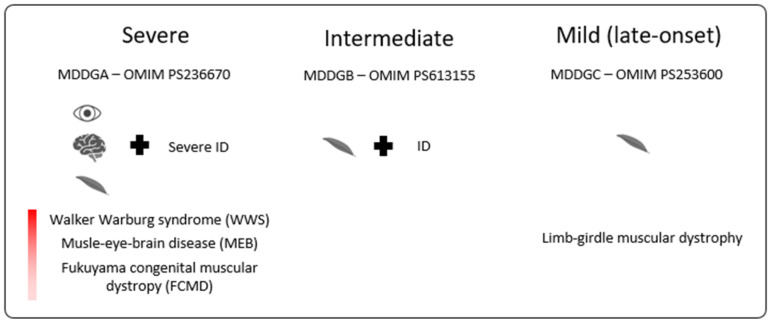
A wide spectrum of clinical severity is observed in patients with dystroglycanopathies. MDDGA is the most severe phenotype and involves patients diagnosed with WWS, MEB, and FCMD based on eye, muscle, and brain anomalies. MDDGB patients have a milder phenotype with muscle and neurodevelopmental delays but without eye or structural brain anomalies. The last group at the mildest end of the spectrum is characterized by limb-girdle muscular dystrophy, mostly seen in late-onset disorders.

Muscular dystrophy-dystroglycanopathies (MDDGs), in short, dystroglycanopathies, result from aberrant glycosylation of α-DG and result in a spectrum of muscular dystrophy that ranges from severe muscle hypoplasia with or without eye and brain anomalies to mild adult-onset limb-girdle muscular dystrophy [5]. As illustrated in Figure 1, MDDGs are classified into three subtypes (A, B, and C) [6]. MDDG type A (MDDGA) is situated at the most severe end of the spectrum and presents with early-onset muscular dystrophy; severe intellectual disability (ID) with brain malformations; and eye involvement with microphthalmia, cataract, and retinal atrophy as described in Walker–Warburg syndrome (WWS, OMIM #236670), muscle-eye-brain disease (MEB, OMIM #236670), and Fukuyama congenital muscular dystrophy (FCMD, OMIM #253800). In MDDG type B (MDDGB), muscular dystrophy associates with ID but without eye or structural brain anomalies. MDDG type C (MDDGC) is limited to (late-onset) limb-girdle muscular dystrophy. Molecular heterogeneity underlies the phenotypic spectrum with the pathogenic variants identified to date in eighteen different genes: *DAG1, POMT1, POMT2, POMGNT1, POMGNT2, LARGE, B4GAT1, B3GALNT2, DPM1, DPM2, DPM3, DOLK, POMK, GMPPB, FKTN, FKRP, ISPD*, *and TMEM5.*

MDDGA11 is caused by biallelic pathogenic variants in *B3GALNT2* (OMIM #610194), encoding β-1,3-N-acetylgalactosaminyltransferase 2. This transmembrane protein resides in the endoplasmic reticulum and, together with O-mannose β1,4-GlcNAc transferase (*POMGNT2*), phosphorylates O-mannosyl trisaccharide [N-acetylgalactosamine-β3-N-acetylglucosamine-β4-(phosphate-6-)mannose] on α-DG. This final trisaccharide is necessary for the high-affinity binding of α-DG to laminin-G domains in extracellular matrix proteins in muscle and the brain [7]. Hitherto, 23 patients with biallelic missense and/or truncating variants in *B3GALNT2* have been identified [8,9,10,11,12]. Though initially associated with MDDGA11 (OMIM #615181), a later report identified patients with mild-to-moderate ID and behavioral problems that may be associated with epilepsy, but without any apparent muscle or eye involvement, or abnormalities on neuroimaging [10].

Here, we report a 7-year-old girl with a homozygous frameshift mutation in *B3GALNT2*, presenting with isolated global developmental delay and central nervous system abnormalities initially attributed to perinatal asphyxia. The homozygous state of the identified *B3GALNT2* variant results from a mixed uniparental disomy (mixUPD) of chromosome 1. This report confirms that whole-exome sequencing (WES) is able to detect uniparental disomy (UPD) [13]. Furthermore, we provide an overview of all previously reported cases highlighting that β-1,3-N-acetylgalactosaminyltransferase 2 deficiency may be associated with milder phenotypes.

## 2. Materials and Methods

### 2.1. Patient Cohort

We reviewed the data of 23 previously reported individuals with *B3GALNT2* defects for whom sufficient clinical data were reported. A detailed overview of the phenotypic features of the patients, including our patient, is shown in Table S1.

### 2.2. Exome Sequencing (ES)

gDNA from whole blood was extracted using the ReliaPrepTM Large volume HT gDNA Isolation System from Promega according to the kit’s instructions. After extraction, ES was performed by enriching the coding exons with the SureSelect All Exon v6 kit (Agilent Technologies, Santa Clara, CA, USA), followed by paired-end 2 × 150 bp sequencing on a HiSeq3000 platform (Illumina, San Diego, CA, USA). Raw sequence reads were processed using an in-house-developed pipeline, and data analysis was limited to a panel of 1109 selected genes associated with intellectual disability and epilepsy. Confirmation and segregation analyses were performed using PCR amplification and Sanger sequencing. Primers are available on request. Since several variants on chromosome 1 were present in the homozygous state, PLINK software was used to detect the regions of homozygosity (ROH) [14].

### 2.3. Array-CGH

Array comparative genomic hybridization (array-CGH) was performed on a 180 k Agilent oligonucleotide array as previously described [15].

### 2.4. SNP-Array

The human CytoSNP-12 BeadChip (Illumina) kit was used for SNP-array (~300,000 SNPs targeting regions). Data analysis at a genome-wide resolution of ±180 kb was performed using the program CNV-WebStore 2.0 (NCBI build hg19/GRCh37).

## 3. Results

### 3.1. Clinical Description

The female proband is the first child born to a non-consanguineous couple after in vitro fertilization and an uncomplicated pregnancy and delivery. The family history was notable for the preterm birth of a paternal half-sister and half-brother at 31 and 35 weeks of gestation, respectively. Both had normal neurodevelopment.

At birth, the proband’s weight was 3110 g (p25), length 49 cm (p25), and head circumference 32.5 cm (p3). There were no neonatal problems apart from low intake but with normal staturoponderal evolution. She has global developmental delay. She crawled at age 18 months, and she could walk independently at 27 months. She spoke her first words at 20 months, but, at age 4, she still only used about 10 words. She learned simplified sign language. Her passive language development was better. Her developmental age was 25 months for a calendar age of 4 years and 3 months. She further showed the tendency of an autism spectrum disorder, a high pain threshold, and balance problems.

A physical examination at age 7 years and 4 months showed a height of 128 cm (p70), a weight of 27 kg (p72), a head circumference of 50 cm (p16), blond curly hair, upslanted palpebral fissures, light blue irises, epicanthal folds, fullness of the upper eyelids, a tubular nose with a broad nose tip, and hypoplastic alae nares (Figure 1). She had a small auricular tag on the helical crus and large ear lobes. She had widely spaced nipples, slender hands with rather long fingers, and slightly prominent wrinkles of the skin.

**Figure 2:**
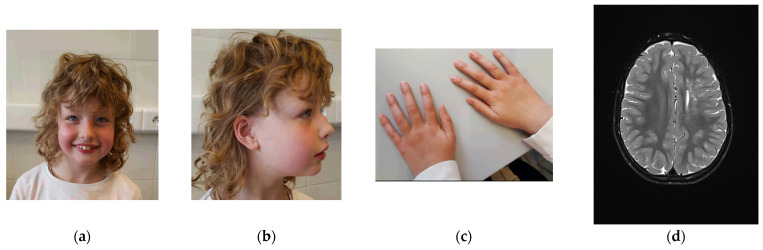
Physical examination at age 7 years and 4 months. (**a**) Facial features include upslanting palpebral fissures, light blue irises, epicanthal folds, fullness of the upper eyelids, a tubular nose with broad nose tip, and hypoplastic alae nares. (**b**) Side view: small auricular tag on the right ear and large ear lobes. (**c**) Picture of the hands: slender hands with rather long fingers, and slightly increased skin wrinkling. (**d**) Brain magnetic resonance imaging (MRI) at age 6 years and 9 months shows an increased T2 signal in the corona radiata on the left side. Furthermore, limited ex vacuo dilatation of the left lateral ventricle and atrophy of the anterior segment of the corpus callosum are present.

Orthopedic evaluation suggested retained anteversion of the hips with internal rotation of the limbs as a cause for frequent stumbling, for which physiotherapy was initiated. Echocardiographic evaluation was normal. Eye examination was normal, apart from intermittent esophoria, which spontaneously disappeared. Her hearing was normal (latest evaluation at age 4). Brain magnetic resonance imaging (MRI) at ages 3 years and 6 months and at 6 years and 9 months (Figure 2) showed an increased T2 signal in the corona radiata on the left side, expanding to the lateral ventricular wall with sparing of the juxta cortical white matter, as well as bilaterally frontally. There was limited ex vacuo dilatation of the left lateral ventricle and atrophy of the anterior segment of the corpus callosum. These findings remained stable over time. There was slight cerebellar hypotrophy. An initial diagnosis of perinatal asphyxia was refuted because of a normal perinatal course. A metabolic screening with serum amino acids, lactate, pyruvate, acylcarnitines TSH and FT4, long-chain fatty acids, phytanic and pristanic acids, and transferrin isoelectric focusing was normal.

### 3.2. Molecular Result

**Figure 3:**
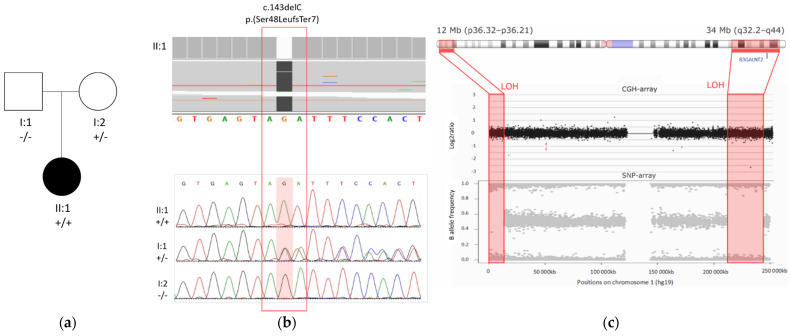
*B3GALNT2* variant and mixed UPD found by WES. (**a**) Pedigree of the family of the patient described in this case report; the mutant allele is represented with a ‘+’ sign and the wild type allele with a ‘–’ sign. (**b**) Top: exome sequencing reads of the proband (II:1) showing the homozygous deletion variant c.143delC in *B3GALTN2* (NM_152490.4) (Hg38). Bottom: segregation analysis of this variant showed a homozygous state in the proband, a heterozygous state in the mother, and absence in the father. (**c**) Loss of heterozygosity (LOH) in two regions on chromosome 1, detected by ES, was confirmed by SNP-array; array-CGH excluded a deletion. *B3GALNT2* is located in the largest LOH region (q32.2–q44).

WES detected two homozygous regions of approximately 12 Mb and 34 Mb, separated by a 200 Mb region on chromosome 1 (Figure 3). Within the largest homozygous region, a homozygous frameshift variant was identified in *B3GALNT2* (NM_152490.4): c.143delC; *p*.(Ser48LeufsTer7). This frameshift variant resides in exon 2, is absent in the population database gnomAD, and has not been reported previously. The variant likely causes nonsense-mediated decay (NMD). Genomic microarray analysis (array-CGH) did not reveal any significant copy number variants.

The variant is present in a heterozygous state in the mother and is absent in the father. Paternity was confirmed, and a paternal deletion on chromosome 1 was excluded by array-CGH. Therefore, the large interspace of heterozygous variants between the two homozygous regions was suggestive of mixed UPD, which was confirmed by SNP-array analysis determining the size of the homozygous regions to be 12 Mb and 34 Mb located at p36.32–p36.21 and q32.2–q44 on chromosome 1, respectively (Figure 3).

### 3.3. Review of Previously Reported Cases

Table 1 gives an overview of all the reported individuals with (likely) pathogenic variants in *B3GALNT2*, totaling 23 cases, including ours (patient P23). Three groups can be clinically distinguished as follows: group 1 represents the most severe phenotype, presenting with WWS; group 2 presents an intermediate phenotype, with MEB or FCMB; and group 3 presents atypical CMD with no obvious muscular anomalies. Both groups 1 and 2 are categorized as MDDGA in OMIM. See Table S1 for a more detailed overview of the phenotype.

Individuals 1–15 present with clinical features of MGGDA, while patients 16–23 present with mild-to-moderate ID without major eye, muscle, or brain involvement. All show intellectual disability and motor delay with variable severity. Except for individuals for whom no information on language development is available, all present with severe active language delay with absent speech or speech limited to single words or sign language (17/17). Epilepsy occurs in about half of all of the probands (8/21), but it does not correlate with the severity of the ID. Hypotonia is observed in 10 out of 18 individuals. Some patients (10/13) present with additional neurological symptoms, including ataxia, spasticity, and balance problems. Ocular involvement occurs in ten cases and includes optic nerve hypoplasia, microphthalmia, cataract, esotropia, and blindness. Speech and motor development seem to be better in patients without ocular involvement.

In all assessed individuals, brain MRI shows clear abnormalities. At the severe end, patients with WWS (P1–P5) show cobblestone lissencephaly and hydrocephaly. In contrast, patients with isolated ID show increased T2 hyperintensities of the white matter, sometimes accompanied by cerebellar hypoplasia. These changes have to be differentiated from the leukoencephalopathy seen following peripartal asphyxia. Notably, the presence of cerebellar anomalies is high (18/21) and may present as pontocerebellar hypoplasia. In patients 20, 21, and 23, a Computed Tomography (CT) scan failed to reveal any abnormalities, which likely reflects the lower sensitivity of CT compared to MRI.

**Figure 4:**
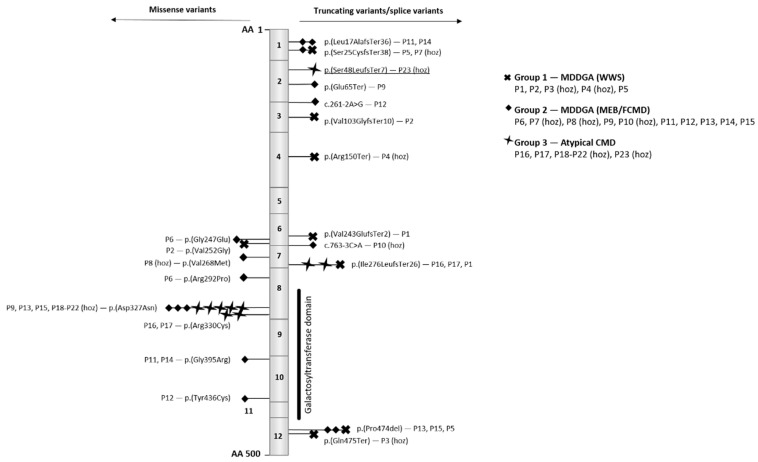
Overview of the *B3GALNT2* (NM_152490.4) gene and the reported variants. The gene consists of 12 exons and encodes a 500 amino acid (AA) protein. The missense variants are positioned on the left and the truncating on the right. As illustrated in the legend, mutations are marked by a symbol depending on patient phenotype. For each mutation, the identification number of the patient is mentioned (see also Table 1), and homozygosity is indicated by (hoz). Patient 23 is underlined and refers to the patient discussed in this paper.

Both missense and truncating *B3GALNT2* variants have been identified. Truncating variants are spread throughout the entire gene, while missense variants are enriched in exons 6, 7, 8, and 10 (Figure 4). Exons 8 and 10 encode part of the galactosyltransferase domain. Four pathogenic missense variants have been identified in this domain, including the recurrent p.(Asp327Asn) variant. In one family, individuals P18–P22, all homozygous for p.(Asp327Asn), have intellectual disability, motor delay, and epilepsy but do not show any remarkable muscular problems, which is in contrast to patients P9, P13, and P15, who do exhibit muscular problems and are compound heterozygous for this variant and the p.(Glu65Ter)(in P9) or the p.(Pro474del) variant (in P13 and P15).

## 4. Discussion

We describe a 7-year-old girl with global developmental delay, severely delayed active language development, and autism spectrum disorder as a result of a homozygous truncating variant in *B3GALNT2* due to a mixed uniparental disomy of chromosome 1. Exome sequencing identified two homozygous regions interrupted by a large heterozygous region, which is likely explained by recombination during meiosis I or II and by trisomic rescue [16]. This confirms that ES data are suitable to detect uniparental disomy, which may be an important and sometimes overlooked cause of Mendelian disease [13].

Our case further demonstrates that the phenotypic spectrum due to *B3GALNT2* pathogenic variants may present with a milder disease phenotype, limited to isolated ID, without eye or major muscle involvement [10]. Individuals at the mild end of the spectrum may show white matter anomalies, with or without cerebellar hypoplasia, but no major brain malformations.

It has been suggested that bi-allelic loss-of-function variants cause a severe phenotype of WWS [10]. Nevertheless, the data show that truncating variants predicted to result in nonsense-mediated decay may cause disease at both ends of the spectrum. Indeed, our case (individual 23) illustrates that a homozygous frameshift mutation *p*.(Ser48LeufsTer7) may present with isolated ID. Moreover, WWS has been associated with a nonsense variant at codon 475 (individual 3) residing in the last exon of the transcript and is therefore expected to escape nonsense-mediated decay with preservation of the functional domain. Therefore, the severity of the symptoms cannot be fully explained by the position or by the type of mutation. To date, homozygosity for *p*.(Asp327Asn) has been associated with a milder phenotype; however, all patients belong to the same family, and other genetic modifiers of the phenotype may have been present. To date, no glycosylation staining of α-dystroglycan has been performed in patients with mild disease.

## 5. Conclusions

This study confirms that *B3GALNT2* pathogenic variants may result in a milder disease than initially reported, confined to isolated intellectual disability. No clear genotype–phenotype correlations can be identified for *B3GALNT2*-related dystroglycanopathy. In addition, we show that ES is well suited to identify uniparental disomy.

## Figures and Tables

**Table 1:** Overview of patients with a mutation in *B3GALNT2*.

Patient Number	c. and p. Position of Variant	Type Variant	Phenotype
P1	c.726_727del; p.(Val243GlufsTer2) c.822_823dup; p.(Ile276LeufsTer26)	Frameshift Frameshift	WWS (MDDGA)
P2	c.308_309del; p.(Val103GlyfsTer10) c.755T > G; p.(Val252Gly)	Frameshift Missense	WWS (MDDGA)
P3	c.1423C > T; p.(Gln475Ter)	Nonsense (hoz)	WWS (MDDGA)
P4	c.448C > T; p.(Arg150Ter)	Nonsense (hoz)	WWS (MDDGA)
P5	c.51_73dup; p.(Ser25CysfsTer38) c.1421_1423delCTC; p.(Pro474del)	Frameshift In-frame	WWS (MDDGA)
P6	c.740G > C; p.(Gly247Glu) c.875G > C; p.(Arg292Pro)	Missense Missense	MEB/FCMD (MDDGA)
P7	c.51_73dup; p.(Ser25CysfsTer38)	Frameshift (hoz)	MEB/FCMD (MDDGA)
P8	c.802G > A; p.(Val268Met)	Missense (hoz)	MEB/FCMD (MDDGA)
P9	c.192dupT; p.(Glu65Ter) c.979G > A; p.(Asp327Asn)	Nonsense Missense	MEB/FCMD (MDDGA)
P10	c.763-3C > A	Splice acceptor (hoz)	MEB/FCMD (MDDGA)
P11	c.48dup; p.(Leu17AlafsTer36) c.1183G > A; p.(Gly395Arg)	Frameshift Missense	MEB/FCMD (MDDGA)
P12	c.261-2A > G c.1307A > G; p.(Tyr436Cys)	Splice donor Missense	MEB/FCMD (MDDGA)
P13	c.979G > A; p.(Asp327Asn) c.1421_1423delCTC; p.(Pro474del)	Missense In-frame	MEB/FCMD (MDDGA)
P14	c.48dupG; p.(Leu17fsTer36) c.1183G > A; p.(Gly395Arg)	Frameshift Missense	MEB/FCMD (MDDGA)
P15	c.979G > A; p.(Asp327Asn) c.1421_1423delCTC; p.(Pro474del)	Missense In-frame	MEB/FCMD (MDDGA)
P16	c.822_823dup; p.(Ile276LeufsTer26) c.988C > T; p.(Arg330Cys)	Frameshift Missense	Atypical CMD
P17	c.822_823dup; p.(Ile276LeufsTer26) c.988C > T; p.(Arg330Cys)	Frameshift Missense	Atypical CMD
P18	c.979G > A; p.(Asp327Asn)	Missense (hoz)	Atypical CMD
P19	c.979G > A; p.(Asp327Asn)	Missense (hoz)	Atypical CMD
P20	c.979G > A; p.(Asp327Asn)	Missense (hoz)	Atypical CMD
P21	c.979G > A; p.(Asp327Asn)	Missense (hoz)	Atypical CMD
P22	c.979G > A; p.(Asp327Asn)	Missense (hoz)	Atypical CMD
P23 (our case)	c.143del; p.(Ser48LeufsTer7)	Frameshift (hoz)	Atypical CMD

Abbreviations: CMD, congenital muscular dystrophy; FCMD, Fukuyama congenital muscular dystrophy; hoz, homozygous; MEB, muscle–eye–brain disease; MDDGA, muscular dystrophy-dystroglycanopathy type A; WWS, Walker–Warburg syndrome.

## Data Availability

Not applicable.

## Supplementary Materials

The following supporting information can be downloaded at: https://www.mdpi.com/article/10.3390/genes13040694/s1, Table S1: Overview of *B3GALNT2* mutations.
